# Super Wide Band, Defected Ground Structure (DGS), and Stepped Meander Line Antenna for WLAN/ISM/WiMAX/UWB and other Wireless Communication Applications

**DOI:** 10.3390/s20061735

**Published:** 2020-03-20

**Authors:** Shahid Ullah, Cunjun Ruan, Muhammad Shahzad Sadiq, Tanveer Ul Haq, Ayesha Kosar Fahad, Wenlong He

**Affiliations:** 1 School of Electronic and Information Engineering, Beihang University, Beijing 100191, China; shahidkhan@buaa.edu.cn (S.U.); shahzadsadiq@buaa.edu.cn (M.S.S.); anveerulhaq@buaa.edu.cn (T.U.H.); ayeshakosar@buaa.edu.cn (A.K.F.); 2 Beijing Key Laboratory for Microwave Sensing and Security Applications, Beihang University, Beijing 100191, China; 3 College of Electronics and Information Engineering, Shenzhen University, Shenzhen 518060, China; wenlong.he@szu.edu.cn

**Keywords:** bandwidth ratio, DGS, stepped meander line, super wide band, time-domain analysis

## Abstract

This paper presents a new shape (s-shape monopole) of a super wideband antenna using stepped meander lines, a quarter waveguide transformer feeding line, and a defected ground structure (DGS). The antenna will be used for multiple wireless communication applications like WIMAX/WLAN/ISM/UWB, and also for several wireless communication applications. The total dimensions of the proposed antenna are 35 mm × 35 mm × 1.57 mm or 0.36 λo × 0.36 λo × 0.016 λo, which are the corresponding electrical dimensions with free-space wavelength (λo) at the lower operating frequency. The antenna is designed and simulated into two steps: the first (Antenna 1) covers a bandwidth of 18.2 GHz, while the second (Antenna 2, using DGS) covers a super wide bandwidth of 37.82 GHz (3.08–40.9 GHz). The measured fractional bandwidth and bandwidth ratio of the antenna are 174.68% and 13.009:1, respectively, which is operating from 3.09–40.2 GHz. The maximum calculated gain and efficiency are 5.9 dBi and 92.7%, respectively. The time-domain performance is good due to the calculation of the system fidelity factor, group delay, and its linear and constant phase variation.

## 1. Introduction

Monopole antennas are favored in wireless communication applications due to their wide bandwidth, small size, design simplicity, and ease of integration with the other devices [1]. Such wideband antennas are very useful for lung cancer and microwave imaging [2,3]. In 1976, Dubost and Zisler introduced a wideband application monopole antenna [4]. Among other types of antennas, monopole antennas well known because of their suitable applications, i.e., Bluetooth, wireless USB dongle, satellite communication, HIPERLAN/2, and WIMAX [5]. Researchers have designed and implemented different types of monopole antenna structures to achieve wide bandwidth, e.g., U-shape monopole [6], octagon shape [7], printed T-shape [8], spline-shaped monopole [9], Koch fractional shape [10], FTSE (folded t-shaped monopole) [11], and trident shape strip-feed monopole [12]. Moreover, researchers have used various techniques to achieve super wide bandwidth, such as defected ground structures where different types of slots were used in the ground plane of the antenna. Such designs consist of tapered shape slots [13], hexagonal slots [14], and T-shape slots [15]. In microstrip antennas, DGS is used for the enhancement of gain and bandwidth. Also, it has been used for higher mode harmonic suppression, mutual coupling between elements, and to improve the characteristics of the microstrip antenna radiation. The second method is to change the feeding line; it uses a suitable feeding line for proper impedance matching over a broad range of frequencies to achieve wide bandwidth. Some examples of this are the antipodal Y-strip [16], dual orthogonal microstrip feeding lines [17], and an inverted F-feed line [18]. The third technique is the use of meander line structures that are suitable for wide bandwidths due to their mutual coupling mechanism. This type of structure is used to miniaturize antennas. Its different parts operate at different frequencies [19].

Nowadays, the demand for super-wide band antennas for both long- and short-range transmissions for future ultra-wide band communication applications is very high. The term SWB (super-wideband) refers to bandwidth ratios of 10:1 or higher at a 10 dB return loss [20]. Many SWB antennas for advanced wireless communication in civilian and military systems are presented in the literature. Recently, several monopole antennas have been designed which achieve wide bandwidths. One recent research work presented a monopole antenna having a wide bandwidth. The antenna achieved 86% radiation efficiency and 153.22% fractional bandwidth, and its applications included WLAN, WiMAX, ISM, and wireless communication [21]. In [22], a monopole antenna was implemented for WPAN (wireless personal area network) with a 79.21% radiation efficiency and a 135.2% fractional bandwidth. In [23], slotted antennas were designed with a microstrip feeding line, and the author achieved 138% bandwidth and 88% radiation efficiency. In [24], a circular antenna using a waveguide transformer feeding line was presented. It achieved a super wide bandwidth of 165.7% (2.7–28.8 GHz). In [25], a super wide band antenna is presented; it achieved a 133% (10–50 GHz) bandwidth by using an octagonal fractal microstrip patch. A propeller shape was used by replacing the circular radiating shape of the monopole antenna to achieve a 168% (3–35 GHz), operating bandwidth [26]. These examples show that with the help of monopole antennas, super-wide bandwidths can easily be achieved by using multiple techniques.

This paper describes an S-shape stepped meander line monopole antenna with a defected ground structure which is capable of operating at a super-wide bandwidth. The simulated operating frequency of the antenna is from 3.08 GHz to above 40.9 GHz, and the measured frequency range is from 3.09 GHz to 40.2 GHz. A quarter waveguide transformer feeding line is used for best matching and to achieve a wide bandwidth.

Two antennas were tested with side by side and face to face alignments to check the performance in the time domain. We observed acceptable results regarding the group delay and system fidelity factor. The structure of this paper is as follow: in Section 2, the design and configuration of the antenna is presented: Section 3 consists of a parametric study of the DGS slot; in Section 4, and antenna simulation and measurement results are discussed; Section 5 describes the time-domain performance of the antenna; Section 6 presents a comparison of the results of the proposed work with previous works; finally, Section 7 presents the conclusion.

## 2. Antenna Design and Configuration

**Figure 1:**
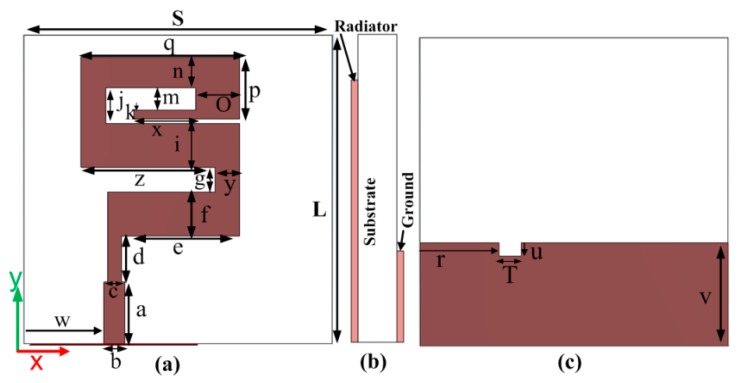
Proposed antenna (**a**) front view (**b**) side view (**c**) back view.

**Figure 2:**
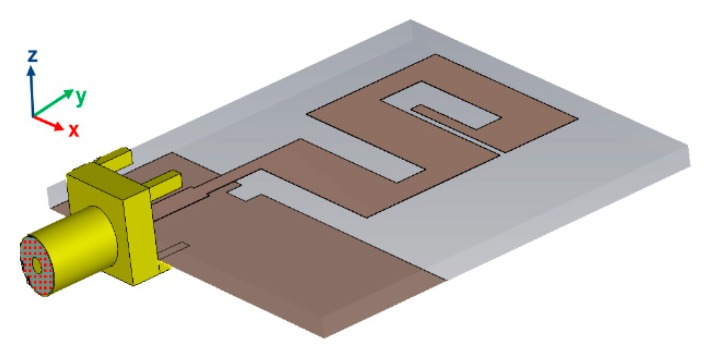
3D view of the proposed antenna.

The antenna described in this paper was designed on a Roger 5880 substrate having permittivity (ε_r_ = 2.2), tangent loss 0.0009, and a thickness of 1.57 mm. A perfect electric conductor (PEC) having 0.035 mm thickness was used to simulate the radiator element, microstrip line, and ground of the antenna. The proposed design of the antenna with front, side, and back views is shown in Figure 1a–c. A 3D view of the proposed antenna is shown in Figure 2. According to the monopole antenna configuration, the length and width were calculated with the help of λ/4 and λ/100 respectively [8], where “λ” is the wavelength of the antenna at the center frequency. Table 1 shows the dimensions of all the parts of the antenna. The proposed structure is different from that of simple meander lines. Every rod of the antenna operates at different frequencies because the frequency of the antenna depends on the corresponding wavelength. When the wavelength is small, the frequency will be higher, so the dimensions of the antenna rods are optimized according to the corresponding frequencies. In proposed structure, length “e” is different from lengths “z”, “q”, and “x”; width “n” is different from widths “i”, “k”, and “f”, and the space between the meander lines are not same, but rather, are optimized to get a wide bandwidth. The meander line rod with length “e” and width “f” operates at higher frequencies. Length “z” and width “i” operate at the middle frequencies, and lengths “q” and “x” and widths “n” and “k” operate at lower frequencies. Spaces “m”, “j”, and “g” play an important role, i.e., mutual coupling at lower and higher bands to get a wide bandwidth. A special type of microstrip line was used, known as a quarter-wave transformer, where the feeding line is divided into two parts, as shown in Figure 1a. In this figure, line d is known as the transition line, which connects the radiating meander lines with an impedance of Z_L_ = 115.74 Ω, with a Zo = 70 Ω microstrip line. The transition line impedance “Z_T_”, transition line width “c”, and length “a” calculations are explained in [27] and [28].

**Figure 3:**
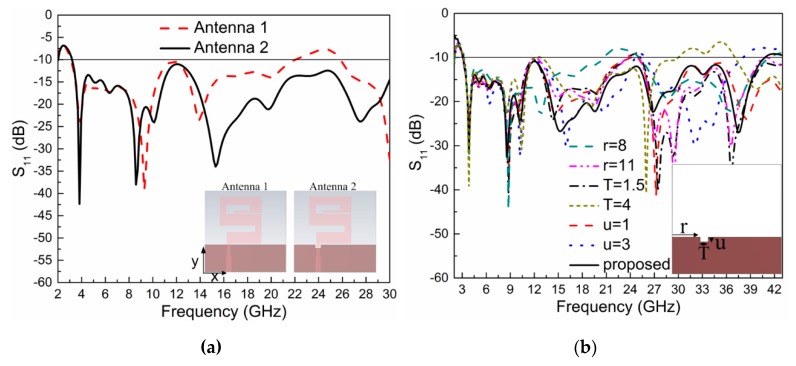
(**a**) S_11_ (dB) results comparison of Antenna 1 without DGS and Antenna 2 with DGS, (**b**) Varition of S11 (dB) with different values of DGS structure width (T), height (u), and its placement from one side of the ground (r).

The length of the transition line at the center frequency (21.65 GHz) is 5.2 mm. The impedance of the transition line is 90.01 Ω and width c = 1.6 mm. The proposed width of the transition line is the optimized value. In the proposed work, two designing steps were used to get a super wideband result. In the first step, a simple meander line antenna (Antenna 1) without DGS was designed and simulated with the same dimensions as those shown in Table 1. The return loss (dB) at this step is shown in Figure 3a, which operates in frequency range of 2. 95 GHz to 21.15 GHz. This antenna covers the S/C/X and Ku frequency bands. To obtain the K-band and Ka-band frequencies ranges, a defected ground structure was used in the second step of antenna design, which is the proposed antenna (Antenna 2), as shown in Figure 3a. The slot in the ground was etched into the backside of the quarter-wave transformer microstrip line. Due to the use of DGS, the antenna now operates above 30 GHz, as shown in Figure 3a.

The physics behind the DGS is as follows: with the help of DGS, the ground plane current distribution will be disturbed. This disturbance will change the transmission line characteristics by including some of the parameters of the slot (capacitance, inductance, and resistance) in the parameters of the transmission line (capacitance, inductance, and resistance). In other words, when the ground plane under the microstrip line is defect, then the microstrip line effective inductance and capacitance will be changed by adding the slot capacitance, inductance, and resistance; therefore, resonance will occur at a certain frequency. The area of the slot is inversely proportionate to the effective capacitance and directly proportionate to the effective inductance. The effective capacitance will reduce due to the decrement of the slot area, and, as a result, the resonance frequency and the effective inductance will increase due to the increase of the slot area. As such, the results will be at the lower cutoff frequency. The aforementioned relationships and DGS circuit diagrams are discussed in paper [29].

## 3. Parametric Study

This section will present a parametric study of the DGS slot width (T), height (u), and the distance (r) of the slot from one side of the ground. The dimensions of the slot affect the bandwidth of the antenna. The proposed parameters (r, T, and u) are shown in Table 1. The slot is etched into the back side of the microstrip line because the microstrip line is used for matching, and its matching bandwidth will be affected by variations in the dimensions of the DGS slot. The distance of the slot from the left side of the ground is denoted by ‘r’; its parametric study results are shown in Figure 3b. This shows that when ‘r’ increases or decreases from its proposed value, the bandwidth of the antenna decreases.

The second parameter is the width of the slot, which is denoted by ‘T’, as shown in Figure 3b. The figure shows that if the width of the slot decreases, the bandwidth will decrease, while when the width of the slot increases, the bandwidth will be also affected.

The third parameter is the height of the slot, denoted by ‘u’, as shown in Figure 3b). The figure shows that if the proposed height (u) decreases or increases, then the resulting bandwidth of the antenna will decrease. When one parameter was changed in the parametric study, the others were kept constant and equal to the proposed values given in Table 1.

## 4. Antenna Simulation and Measurement Result

### 4.1. Simulated Results

The antenna was simulated with the help of the commercial Finite-Integration Technique (FIT) in the electromagnetic simulation software, CST-Microwave Studio-2015. The results were plotted with the help of originLab-2018, a graphing and analysis software. As explained in Section 2, the proposed antenna was designed in two steps. Its S_11_ (dB) results are also explained in that section and are shown in Figure 3a. The parametric study of the defected ground structure (DGS) is explained in Section 3, and its simulated S_11_ (dB) results are shown in Figure 3b. The proposed design results with a simulation setup of 2–43 GHz are shown in Figure 5b.

**Figure 4:**
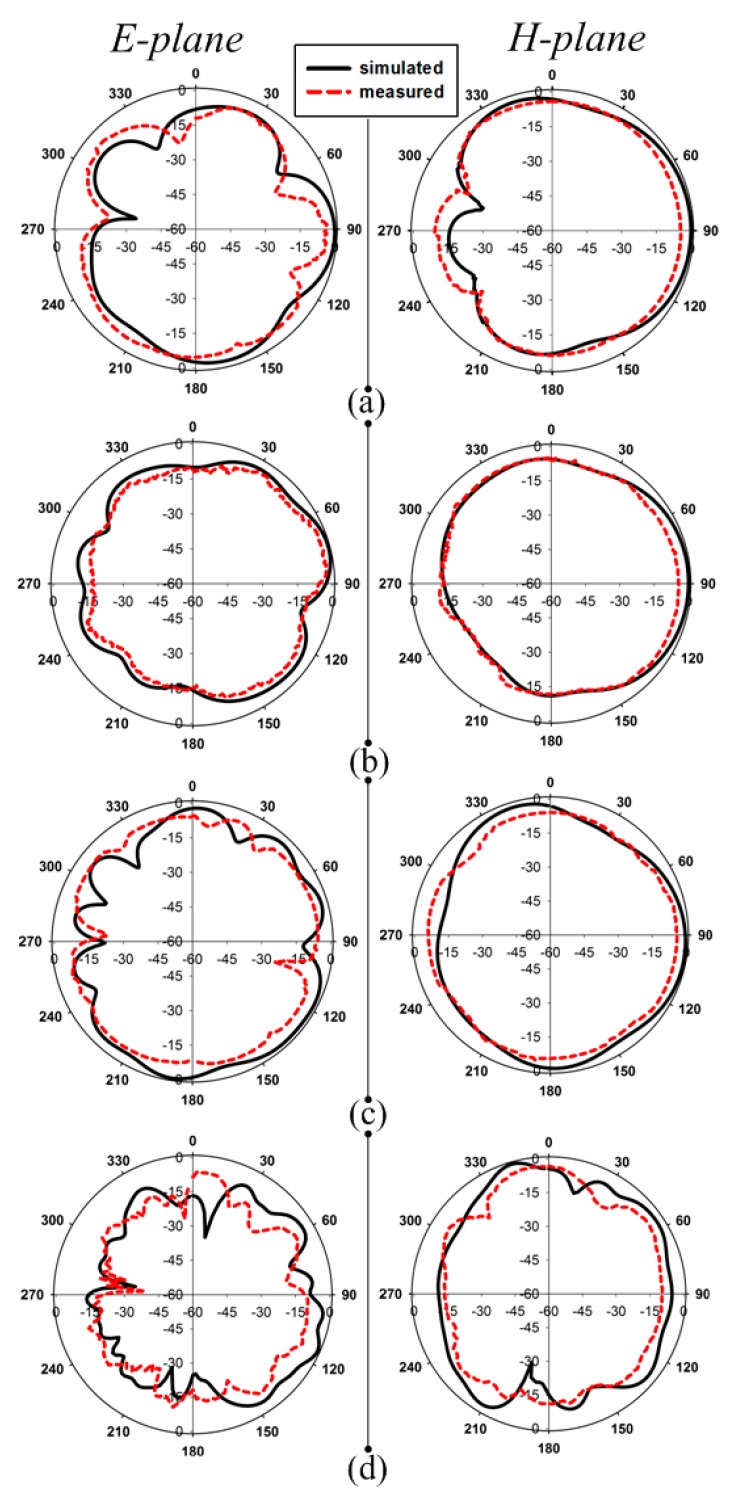
Simulated and measured radiation patterns of the antenna at (**a**) 8.5 GHz (**b**) 12 GHz. (**c**) 18 GHz (**d**) 35 GHz.

**Figure 5:**
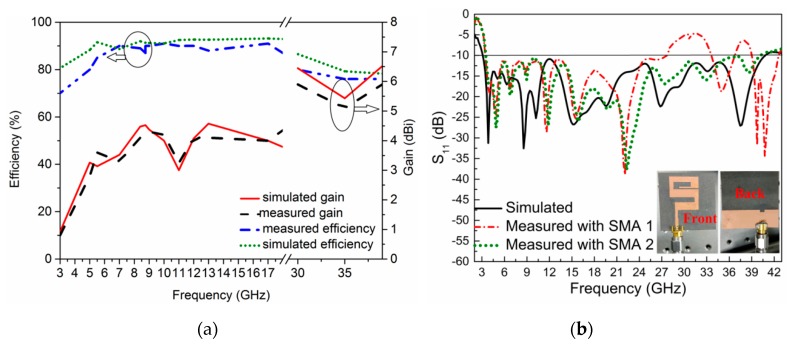
(**a**) Simulated and measured gain and efficiency results of the proposed antenna (**b**) Simulated and measured S_11_ (dB) results with Proposed antenna prototype, front and back side view.

The simulated S_11_ (dB) shows that the antenna covers the S, C, X, Ku, k, and Ka-bands of the microwave range. The proposed antenna is horizontally polarized. The radiation patterns at four frequencies (8.5 GHz, 12 GHz, 18 GHz, and 35 GHz) are shown in Figure 4. The E-plane patterns are in a dumbbell shape, and at some frequencies, higher modes become excited, which may be seen from some of the unwanted ripples at the radiation patterns edges. At lower frequencies, the main lobe of the H-field is in the direction of the y axis, which shows the characteristics of end-fire. Such antennas are best suited to microwave imaging purposes. The patterns also show that the antenna is nearly omnidirectional and suitable for multiple communication applications. The efficiency and gain of the antenna are shown in Figure 5a. According to the simulation, the antenna showed a 95.5% maximum radiation efficiency, and an efficiency of more than 90% across the entire operating band. The simulated maximum gain of the antenna is 6.53 dBi, and the range of the gain at the operating bandwidth is 1.8–6.53 dB.

### 4.2. Antenna Fabrication and Measurement Results

The antenna fabrication is very simple; a thermal transfer method was used. The structure was printed on paper (thermal paper) with the help of CorelDraw or AutoCAD software. Then, the mask was transferred to the substrate (Roger-5880) using a heating machine, the HK320SR.

**Figure 6:**
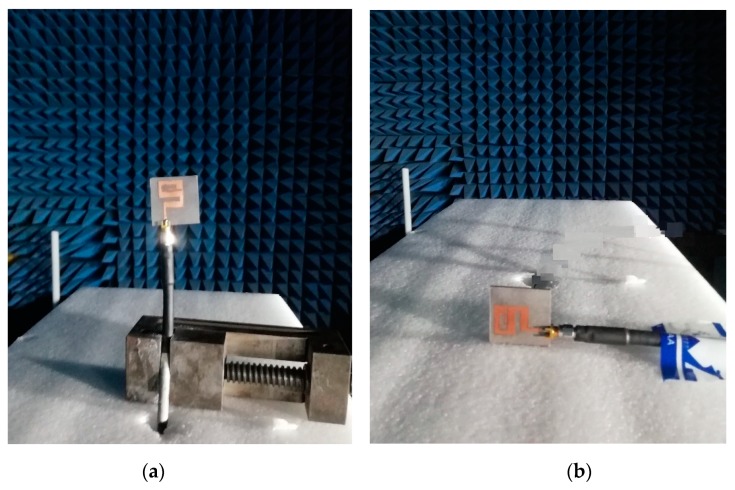
(**a**) Measurment setup for E-plane (**b**) measurement setup for H-plane.

The S_11_ (dB) of the antenna was measured using an AV3672 vector network analyzer (VNA) and the radiation patterns, gain, and efficiency were tested and calculated using an anechoic chamber. The radiation patterns of the antenna are shown in Figure 4. The antenna radiation patterns were tested in two different planes (E- and H-planes). There was some disagreement between the simulated and measured results due to fabrication tolerance. The measured S_11_ (dB) results operatied from 3.09 GHz to 27.55 GHz using an SMA connector (SMA-1 (D550B51H01-118)) and a SMA-2 (SMA2-D360B50H01-118) connector. The S_11_ (dB) operatied from 3.09 GHz to 40.2 GHz, as shown in Figure 5b. The figure shows a frequency shift by measuring using the SMA-1 connector. This shift occurs due to the fabrication tolerance and also to the limited frequency range (27 GHz) of the SMA connector. This connector is still not a suitable candidate for 70 Ω antennas, but there was a small frequency shift and excellent agreement between the simulated and measured bandwidths. At present, we used this special connector (50 Ω) due to the unavailability of a 70 Ω SMA connector. A 13.009:1 bandwidth ratio of the operating frequencies band was shown in the measurement result. The gain and efficiency were calculated, as shown in Figure 5a. The measured gain of the antenna was from 1.6 dBi to 5.9 dBi throughout the operating bandwidth. A maximum measured efficiency of 92.5% was achieved; across the whole frequency range, the efficiency was from 84% to 92. 5%. Due to the wide bandwidth, we measured the proposed antenna gain and efficiency at lower frequencies (3–18 GHz) and at higher frequencies (30–38 GHz), as shown in Figure 5a. The measurement setup for both the E- and H-planes of the anechoic chamber is shown in Figure 6a,b, respectively. In the measurement setup, the transmitted antenna was fixed in front of the antenna under test (AUT).

## 5. Time Domain Performance

A super wide-band antenna which was used to send very short pulses was greatly affected by dispersion. The pulses sent from the transmitting port will never be the same at the receiving port, but the pulses should be recognized by the receiver. For this reason, time-domain analyses are important to calculate the distortion. To this end, two identical antennas were set up with two different orientations (side by side and face to face). In the far-field region, one of the antennas transmits and other receives the signal. An important feature of the antenna is FF (fidelity factor), which compares the transmitted and received signals between two identical antennas. The transmitted and received pulses are normalized, as shown in Equations (1) and (2), respectively. The normalization of the signals was done to compare only the pulse shapes, and not the magnitudes of the signals, because the receiving signal was expected to be much lower than the transmitted one. Between these two signals, cross-correlation was done at every point in time. When overlaps occurred between both pulses, a maximum value of correlation was obtained. Equation (3) was used to calculate the fidelity factor [30].

(1) $$T_{s}^{n}=\frac{T_{s}(t)}{\sqrt{\int_{-\infty }^{\infty }{\left|T_{s}(t)\right|}^{2}dt}}$$

(2) $$R_{s}^{n}=\frac{R_{s}(t)}{\sqrt{\int_{-\infty }^{\infty }{\left|R_{s}(t)\right|}^{2}dt}}$$

(3) $$FF=\max\int_{-\infty }^{\infty }T_{s}^{n}(t)R_{s}^{n}(t+\tau )dt$$

**Figure 7:**
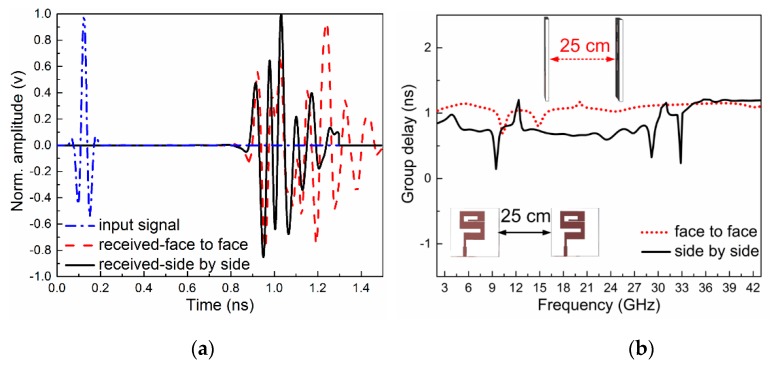
(**a**) Input and received signals for face to face and side by side configuration (**b**) Measured group delay (ns) with two different orientations.

The normalized values of the input and received signal for the side by side and face to face configurations are shown in Figure 7a. The calculated fidelity factor by face to face alignment is 86.7% and for side by side alignment is 82.3%. The second feature of the antenna is to find the group delay, also known as the time delay of the system. This is used to find the phase distortion of the system, which is defined with the help of the following equation [20].

(4) $$GD=-\frac{d\varphi }{d\omega }$$

where “φ” is the phase of S_21_ (transfer function). The group delay of the proposed work for side by side and face to face orientation is shown in Figure 7b, along with the proposed orientation setup. This figure shows that at some frequencies, there are some variations in the group delay, but the values are still less than 1.4 ns for the whole proposed band. The acceptable value of the group delay is up to 3.8 ns [31].

**Figure 8:**
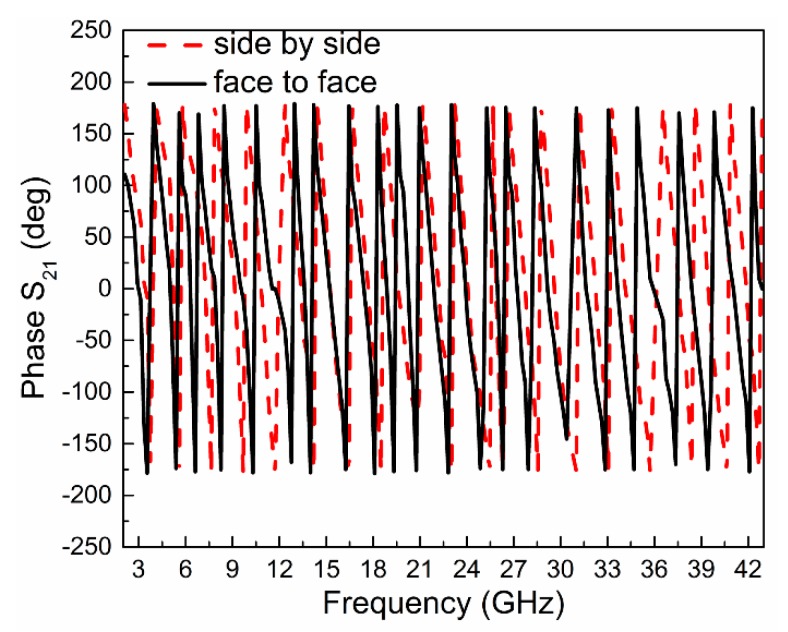
Variation of phase (S_21_) in degree for two different configurations.

The third feature of the antenna is the transfer function where two antennas are connected at the two ports of the vector network analyzer and check the phase of S_21_ in degree. The phase of |S_21_| for two different orientations is shown in Figure 8, which is almost linear and in constant variation, showing that the proposed antenna has less distortion for both configurations (side by side and face to face).

## 6. Comparison

A comparison of the proposed work with previous published similar works having super-wide bandwidths and similar applications is shown in Table 2. The proposed antenna shape (s-shape) is novel; it consists of stepped meander lines, quarter-wave transformer, and the defected ground structure to achieve a super-wide bandwidth. This is the first time that these techniques have been simultaneously applied. The bandwidth of the proposed structure covers all the required bands. The table shows that the proposed antenna has better performance (bandwidth, efficiency, gain, and bandwidth ratio) as compared to those described in the literature. The time-domain analysis of the antenna was good, showing less distortion loss during transmission and receiving.

## 7. Conclusions

This paper presented a new monopole antenna structure that is capable of a super-wide bandwidth, high bandwidth ratio, and high efficiency. The dimensions of the antenna with a high bandwidth ratio (13.009:1) were miniaturized compared to those described in the literature. The objectives were to achieve a super-wide bandwidth by using multiple techniques. This would mean that a single antenna would be suitable for multiple applications, replacing multiple antenna installations and saving antenna real state The structure is very simple in its design and cheap in its fabrication. Due to the end-fire properties of the radiation patterns, the antenna may be utilized in biomedical applications. Due to the satisfactory results (gain, bandwidth ratio, and efficiency) and time domain analysis, the proposed antenna may be used for various wireless communications systems like WIMAX, UWB, WLAN, ISM, and for other multiple defence applications.

## Figures and Tables

**Table 1:** Dimensions of the Proposed Antenna.

Parameters	Values (mm)	Parameters	Values (mm)	Parameters	Values (mm)
a	7	L	35	z	15.2
b	2.4	S	35	W	9.10
c	1.6	n	3.5	j	4
d	5.2	p	8.5	k	1
e	13.4	q	18	x	7
f	5	r	9	m	2.5
i	5	u	1.5	T	2.5
O	5	v	11.7		
g	2.8	y	2.8		

**Table 2:** Comparison of Proposed Work with Previous Work.

Ref. No’s	Bandwidth (%)	Dimension (mm^3^)	Efficiency (%)	Lower Frequency (GHz)	Bandwidth Ratio
[6]	107.35	34 × 20 × 1.6	90	2.27	3.3:1
[10]	122	31 × 28 × 1.6	---	3	4.26:1
[11]	129.24	14 × 18 × 1	---	2.94	4.65:1
[21]	153	25 × 17 × 1.6	86	2.94	7.55:1
[22]	135.2	32 × 32 × 1.6	79.21	2.9	5.17:1
[23]	138	50 × 50 × 1.52	88	2.1	5.47:1
[24]	165.7	30 × 35 × 0.49	96	2.7	10.6:1
[25]	133	60 × 60 × 1.524	---	10	5:1
[26]	168	38 × 55 × 1.6	---	3	11.6:1
[32]	163	40 × 30 × 1.6	75	2.26	9.8:1
[33]	160.4	25 × 35 × 0.83	--	3.5	9.11:1
[34]	168.62	13.4 × 5.2 × 1.6	65	0.4	11.75:1
[35]	150	38.5 × 36.6 × 0.8	90.1	0.55	6.93:1
[36]	166.6	30 × 28 × 1.6	90	3.4	11:1
[37]	164	30 × 45 × 0.175	---	3.15	10.15:1
Proposed work	174.68	35 × 35 × 1.57	92.7	3.01	13.009:1
